# Proteomic insights into COPD pathogenesis and therapeutic targets: a causal analysis of circulating proteins

**DOI:** 10.3389/fmed.2025.1529495

**Published:** 2025-05-12

**Authors:** Min Luo, Ying Tang, Jing Qiu, Kai Yang, WenJing Dai, EnRong Fang

**Affiliations:** ^1^Department of Respiratory and Critical Care Medicine, The First Affiliated Hospital of Chengdu Medical College, Chengdu, Sichuan, China; ^2^School of Clinical Medicine, Chengdu Medical College, Chengdu, Sichuan, China; ^3^Department of Pathology, Clinical Medical College and The First Affiliated Hospital of Chengdu Medical College, Chengdu, China

**Keywords:** chronic obstructive pulmonary disease (COPD), circulating proteins, Mendelian randomization, therapeutic targets, lifestyle modifications, genetic biomarkers, COPD prevention

## Abstract

**Background:**

Chronic obstructive pulmonary disease (COPD) is a preventable and treatable condition, yet current therapies do not halt disease progression, highlighting the need for novel treatments. Using an integrated approach combining proteome-wide Mendelian randomization and Bayesian colocalization analysis, this study identifies novel therapeutic targets for COPD. This study explores causal links between circulating proteins and COPD, identifies potential drug targets, and examines the role of lifestyle factors.

**Methods:**

Using a large proteomics database and a public dataset of 394,244 samples (21,617 COPD cases and 372,627 controls), 4,907 proteins were associated with COPD. Causality was assessed using Bayesian colocalization and proteome-wide Mendelian Randomization (MR), and protein–protein interactions were mapped via STRING. Therapeutic potential was evaluated by identifying drug targets. Additionally, plasma protein expression validation was performed using Western blot experiments in recruited COPD patients and healthy controls.

**Results:**

MR identified 18 proteins linked to COPD, with 11 accelerating disease onset. Strong colocalization evidence was found for MMP12, ASM, KLC1, NPNT and SNX1, none of which overlap with current COPD drug targets. Western blot analysis validated these findings in plasma samples, showing significantly increased expression of MMP12 and ASM, and decreased expression of NPNT and SNX1 in COPD patients compared to healthy controls, while KLC1 showed no significant difference. Notably, MMP12 was negatively associated with grain and nut intake and positively correlated with smoking (*p* < 0.05).

**Conclusion:**

This study identifies potential treatment targets and provides evidence linking specific plasma proteins to COPD risk. Additionally, lifestyle changes may modulate key proteins affecting COPD risk. These findings suggest new avenues for COPD prevention and treatment strategies.

## 1 Introduction

Chronic obstructive pulmonary disease (COPD), a major global health problem, is characterized by persistent respiratory symptoms and limited airflow due to abnormalities in the alveoli as well as airways. These anomalies are typically caused by prolonged exposure to harmful particles or gases (1). One of the top three causes of mortality globally, COPD accounts for about 90% of deaths from the disease in low- and middle-income nations (2). Over 3 million fatalities in 2012 were related to COPD, or 6% of all deaths worldwide. The burden of COPD is predicted to increase dramatically in the ensuing decades because of an aging population and ongoing exposure to risk factors, placing a huge cost on the global economy (3). Thus, early and precise diagnosis and prompt action are essential for reducing the impact of this curable and treatable illness.

COPD is a complex disease influenced by multiple factors. Although smoking is the primary risk factor, genetic factors also play a crucial role in susceptibility to COPD (4). In recent years, genome-wide association studies (GWAS) have identified hundreds of genetic variants associated with COPD risk, including genes encoding matrix metalloproteinase 12 (MMP12), glutathione S-transferase, the alpha-nicotinic acetylcholine receptor, and hedgehog interacting protein (HHIP) (5, 6). However, it remains unclear whether these genes are direct causative agents of COPD or merely markers of other causal genes (7–11). Furthermore, genetic variants can influence protein levels through post-transcriptional processes such as protein stability, translation, secretion, and/or detection of gene products. To date, only a few studies have focused on the impact of genetic variants on a large number of protein biomarkers in chronic diseases. A recent study by Battle et al. highlighted that variations affecting gene expression and protein levels may differ, underscoring the importance of identifying protein quantitative trait loci (pQTLs) to explore the genetic characteristics of disease-related biomarkers. With the rapid advancement of proteomics, large-scale studies have identified over 18,000 protein quantitative trait loci across more than 4,000 proteins (12, 13). These findings provide valuable resources for using Mendelian Randomization (MR) to explore causal relationships between plasma proteins and human diseases, offering new perspectives for uncovering the mechanisms of chronic diseases and identifying potential therapeutic targets.

In addition to genetic factors, environmental effects and lifestyle choices also have a role in the onset of COPD, which typically starts at a young age (14). Hence, encouraging healthy lifestyle choices offers a potential tactic for slowing or stopping the advancement of COPD. Massive studies analyzing blood biomarkers across multiple populations are sparse, despite evidence tying specific blood protein levels to genetic differences (15). Moreover, a thorough investigation of the interaction between genetic markers and disease phenotype prediction is also absent.

To address these gaps, this study performed a proteomic-wide Mendelian randomization (MR) analysis integrated with Bayesian colocalization to investigate the causality between circulating proteins and the risk of COPD. This study uniquely integrates proteome-wide MR analysis with Bayesian colocalization to investigate causal relationships between proteins and COPD, addressing critical gaps in COPD research. This approach aimed to identify novel drug targets and assess potential overlaps with existing therapies. Additionally, this study explored the connection between healthy lifestyle factors and COPD-related proteins, with the goal of identifying key proteins that could serve as intervention targets, thereby facilitating improved prevention as well as management strategies for COPD.

## 2 Methods

### 2.1 Study design

**Figure 1:**
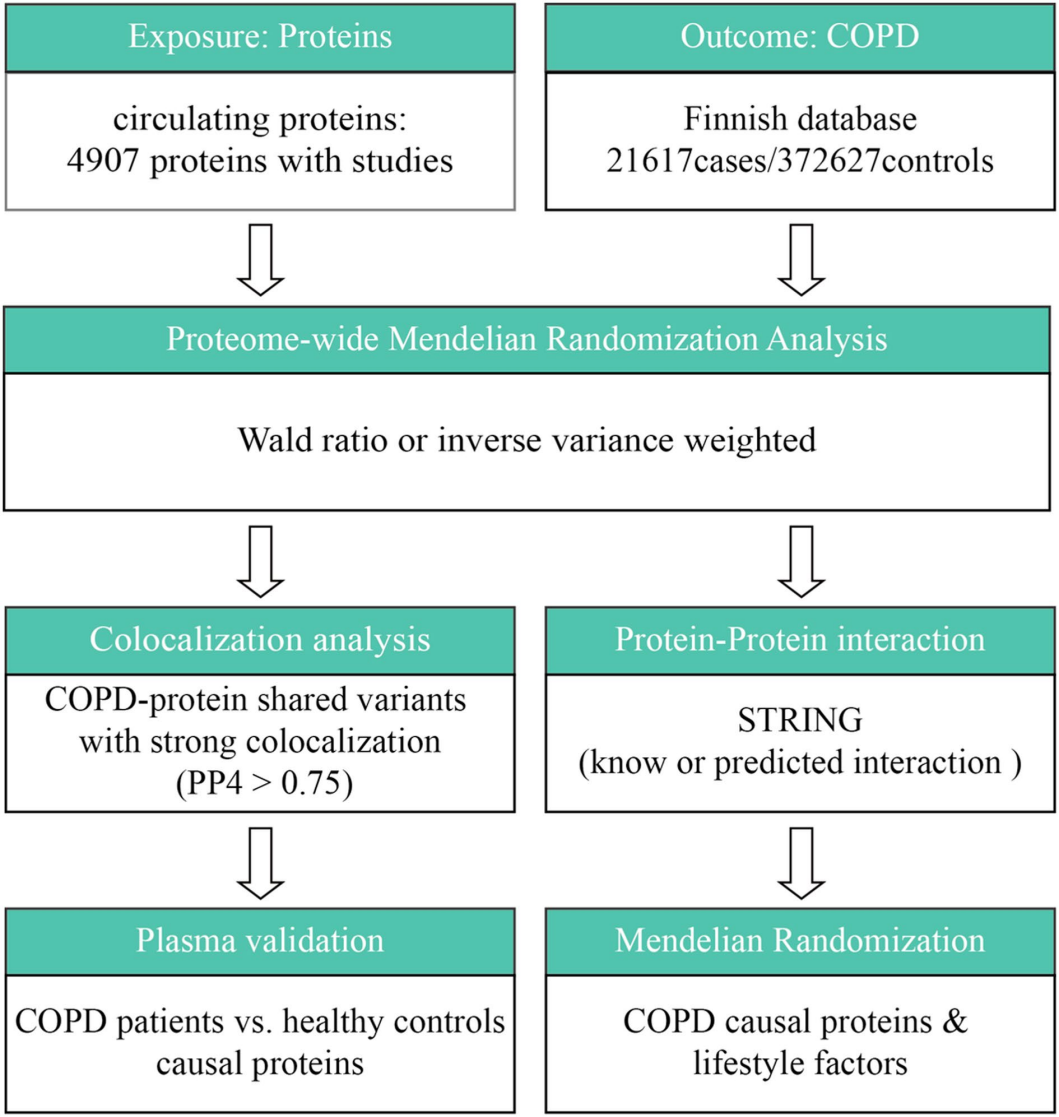
Study design for identification of circulating proteins causally associated with COPD.

The general design of the study is shown in Figure 1. In summary, this study looked at the connection between circulating proteins and COPD via two-sample MR analysis. Cis-pQTL colocalization analysis and GWAS were employed to confirm the causal relationships between circulating proteins as well as COPD susceptibility. Subsequently, this study conducted an investigation of protein–protein interaction (PPI) and evaluated druggability in order to rank possible therapeutic targets. To determine which proteins may serve as interventional targets by healthy lifestyle factors, a thorough MR analysis was performed between COPD-related proteins and healthy lifestyle factors.

### 2.2 Study population and data sources

Using the SOMAscan, summary statistics of genetic correlations for circulating proteins were derived from a large-scale proteomics investigation comprising 35,559 individuals of European ancestry (12). The COPD dataset, encompassing 394,244 cases (21,617 in the experimental group as well as 372,627 in the control group), were obtained from publicly accessible Finnish databases. Participants included adults of European ancestry with high-quality genetic data. Samples with incomplete phenotype data or low genotyping call rates (<95%) were excluded. Quality control for SNPs included Hardy–Weinberg equilibrium (*p* > 1e-6) and minor allele frequency (MAF) > 0.01. Summary statistics for the genetic associations of 17 healthy lifestyle factors were obtained from earlier studies. Every participant gave their informed consent and was of European ancestry. Ethical approval was granted by the appropriate authorities.

### 2.3 Statistical analysis

#### 2.3.1 Proteome-wide MR analysis

The MR analysis relies on three key assumptions (16): (1) genetic variants must influence the exposure (circulating proteins); (2) their effect on the outcome (COPD risk) must occur only through the exposure; and (3) no confounding factors should affect both the variants and the outcome. Instrument strength was assessed using F-statistics (>10), ensuring validity. The formula for the F statistic is as follows: $F=\frac{R^{2}}{1-R^{2}}\times \frac{N-K-1}{k}$, where *R*^2^ is the proportion of variance explained by the instrument, *N* is the sample size, and *k* is the number of instruments. This ensures the reliability of the selected instruments. The analysis comprised measurements of 4,907 circulating proteins from 35,559 people. Proteome-wide MR analysis was undertaken using the TwoSampleMR package. When a protein had only one known instrumental variation, the Wald ratio approach was employed to evaluate associations. A random-effects inverse variance weighted (IVW) technique was employed to proteins represented by genetic instruments to estimate the log-odds change in disease risk per standard deviation rise in protein levels. Statistical power analysis was conducted using the mRnd package to confirm that the study had sufficient power to detect causal associations for the instrumental variables employed. Results showed that all F-statistics exceeded the threshold of 10, indicating strong instrument validity. This method was utilized for proteins having two or more SNP proxies. Sensitivity analyses were carried out to assess the robustness of the results after the original MR analysis using four different methods: weighted mode, weighted median, simple mode, as well as MR-Egger. Multiple testing correction was performed via the Benjamini-Hochberg technique, where a false discovery rate (FDR) of less than 0.05 was deemed statistically significant. The method of mRnd was employed to estimate the statistical power. Reverse MR analysis was also done to investigate the probability of reverse causation. Furthermore, in order to correct for any bias resulting from population stratification in the impact estimations, negative control MR analysis was carried out (17).

#### 2.3.2 Colocalization analysis

In this study, Bayesian colocalization analysis assessed whether identified proteins and COPD share a causal genetic variant. This method differentiates true causal relationships from confounding due to linkage disequilibrium. SNPs within ±500 kb of the pQTL were analyzed, with PP4 > 0.75 indicating robust colocalization (18). Five hypotheses were examined in the analysis:(H0) there is no causal variant for either protein or cancer in the genomic locus; (H1) there is only one causal variant for protein; (H2) there is only one causal variant for protein; (H3) there are two distinct variants for cancer as well as protein; and (H4) the protein, along with cancer shares a causal variant. Default parameters and prior definitions were applied. For each protein, SNPs within ±500 kb of the pQTL were included. In cases where a protein exhibited two and more pQTLs, colocalization analysis was performed on the basis of each pQTL independently, and the most strongly supported pQTL for colocalization was reported. Since colocalization is sensitive to window sizes, a sensitivity analysis based on another window size (±250 kb) was carried out to assess the robustness of the current findings. Robust evidence of colocalization was considered when the posterior probability of H4 (i.e., the hypothesis that the protein and COPD share a causal variant) (PP4) is greater than 0.75 under different windows (19).

#### 2.3.3 PPI and druggability evaluation

The protein–protein interaction (PPI) network was constructed using the STRING database (version 11.5)^1^. The specific parameters were set as follows: the interaction score threshold was set at medium confidence (0.4), and the interaction network was restricted to direct interactions (direct neighbors only). Further, the DrugBank database^2^ was utilized to analyze the drug target potential of candidate proteins, assessing whether current COPD therapeutic agents have direct or indirect interactions with the protein targets identified in this study (20).

#### 2.3.4 Healthy lifestyle factors of COPD causal proteins

In order to identify which lifestyle interventions might be able to control these proteins, this study also used MR analysis to investigate the associations between five COPD-related proteins and healthy lifestyle factors. A total of 17 factors related to a healthy lifestyle were evaluated. These factors included the consumption of sweet cereal, crispbread, other alcohol, herring, cheese, cereal, dried fruit, fruit smoothies, oily fish, milk, water, green tea, length of sleep, consumption of other bread, other meat, cooked vegetables, and status as a non-smoker. The relationships between these factors and the five COPD-related proteins were assessed. The analysis methods used for the MR followed the same procedures as those described in the whole-proteome MR analysis. All statistical analyses were conducted via R software (version 4.1.0).

#### 2.3.5 Patient recruitment and sample collection

This study enrolled 3 patients diagnosed with chronic obstructive pulmonary disease (COPD) and 3 healthy control subjects from March 1 to March 15, 2025, at the First Affiliated Hospital of Chengdu Medical College.

Inclusion Criteria: COPD Group: Participants aged ≥40 years, meeting the Global Initiative for Chronic Obstructive Lung Disease (GOLD) diagnostic criteria for COPD, with a post-bronchodilator FEV₁/FVC ratio <0.70, and no acute exacerbations within the 4 weeks preceding sample collection. Healthy Control Group: Individuals without a history of COPD or other chronic respiratory diseases, free from chronic inflammatory or autoimmune conditions, and with no infections or corticosteroid use in the 3 months prior to sampling.

Exclusion Criteria: Exclusion criteria included a history of malignancy, significant hepatic or renal dysfunction, active infections or systemic inflammatory diseases, immunosuppressant use within the past 3 months, and other chronic respiratory diseases such as asthma, interstitial lung disease, bronchiectasis, tuberculosis, or pulmonary fibrosis.

#### 2.3.6 Western blot analysis

Protein extraction: plasma samples were obtained from the six COPD patients and six healthy controls (designated HC1–HC3 and COPD1–COPD3, respectively). Each 20 μL plasma sample was mixed with lysis buffer containing protease inhibitors, centrifuged at 12,000 × g for 10 min at 4°C to remove debris, and the supernatant was collected for protein concentration determination using the BCA assay.

SDS-PAGE and Western blotting: equal amounts of protein (20 μg per lane) were separated on 10% SDS-PAGE gels and transferred to PVDF membranes. Membranes were blocked with 5% non-fat milk in TBST at room temperature for 1 h, then incubated overnight at 4°C with primary antibodies against MMP12 (abcam, ab52897), ASM (ProteintechGroup, 14,609-1-AP), KLC1 (abcam, ab174273), NPNT (ProteintechGroup, 30,836-1-AP), and SNX1 (abcam, ab134126). After washing, membranes were incubated with HRP-conjugated secondary antibodies at room temperature for 1 h. Signals were detected using ECL chemiluminescence, and band intensities were quantified with ImageJ software. Albumin (abcam, ab207327) served as the loading control.

## 3 Results

### 3.1 Proteome-wide MR identified 18 COPD-related proteins

**Figure 2:**
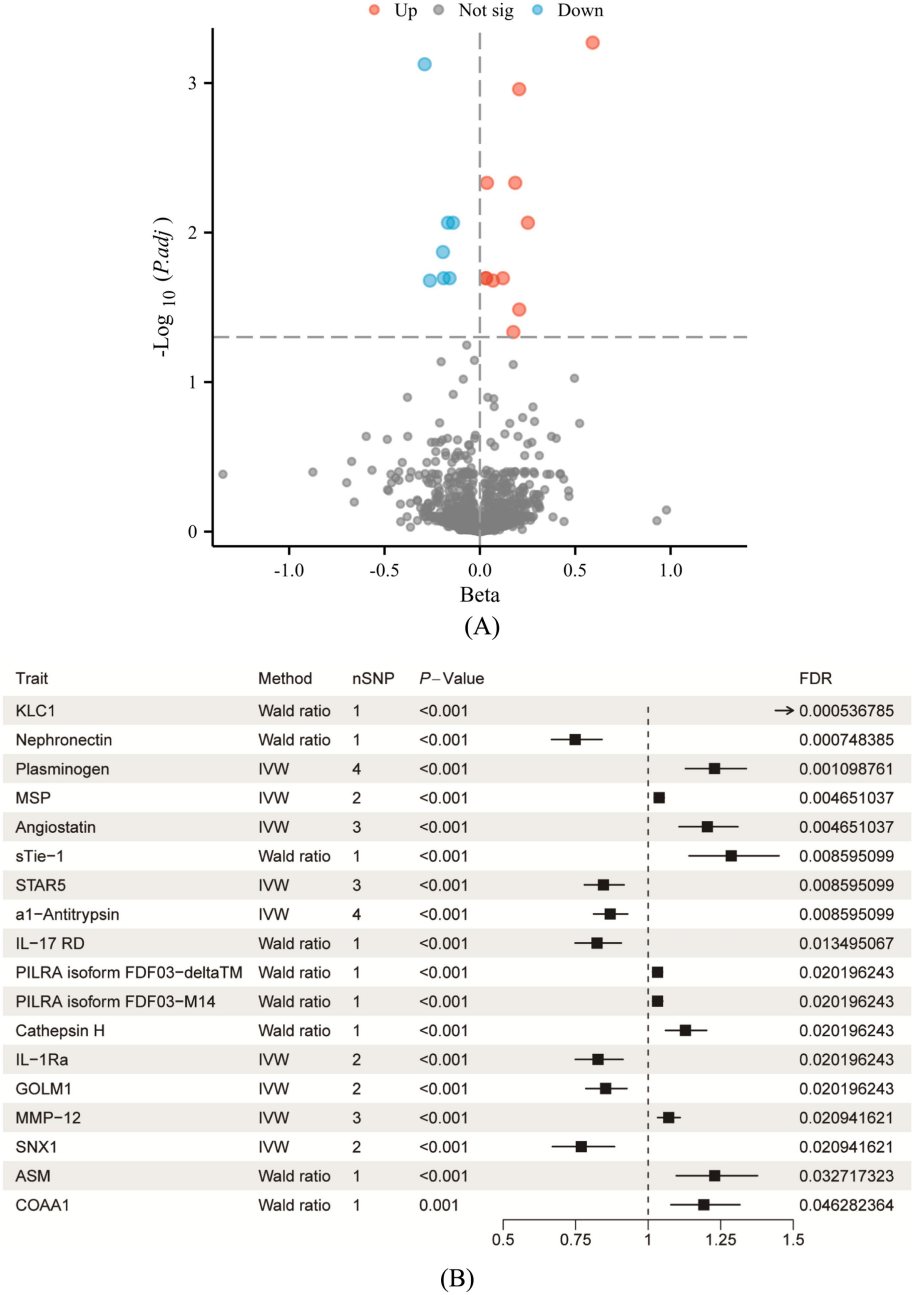
Causal analysis of circulating proteins associated with COPD following FDR correction. **(A)** Volcano plot illustrating the significance and effect sizes of the associations between circulating proteins and COPD. Red dots indicate proteins significantly positively associated with COPD risk, blue dots indicate significantly negatively associated proteins, and gray dots represent proteins that did not achieve statistical significance (*p* > 0.05). **(B)** Forest plot depicting the causal estimates for proteins that remained significant after FDR correction. Distinguishing protective and risk-related effects as potential therapeutic targets. The “Method” column in each row specifies the Mendelian randomization analysis method used; “nSNP” indicates the number of genetic instruments utilized in the analysis; “FDR” indicates the *p*-value adjusted by the false discovery rate.

Through a proteome-wide MR analysis, this study identified 18 circulating proteins that are statistically significantly associated with the presence of COPD (Supplementary Table 1; Figures 2A,B). Among these proteins are sTie-1, STAR5, al-Antitrypsin, IL-7RD, KLC1, Nephronectin (NPNT), Plasminogen, MSP, Angiostatin, PILRA isoform FDF03-deltaTM, PILRA isoform FDF03-M14, Cathepsin H, IL-1Ra, GOLM1, MMP12, SNX1, ASM, and COAA1. In particular, the risk of COPD was found to be decreased by NPNT, STAR5, al-Antitrypsin, IL-17RD, IL-1Ra, GOLM1, and SNX1, and to be increased by KLC1, Plasminogen, MSP, Angiostatin, sTie-1, PILRA isoform FDF03-deltaTM, PILRA isoform FDF03-M14, Cathepsin H, MMP12, ASM, and COAA1. Of these, five proteins—MMP12, ASM, KLC1, NPNT, and SNX1—stand out with strong colocalization evidence (PP4 > 0.75). MMP12 and ASM are implicated in inflammatory processes and tissue remodeling, critical to COPD pathogenesis. NPNT supports cellular adhesion and tissue integrity, while SNX1 maintains cellular trafficking. KLC1 may influence COPD through its role in intracellular transport and cellular homeostasis. There was not any discernible pleiotropy (Ppleiotropy >0.05) or heterogeneity (Pheterogeneity >0.05).

### 3.2 Five proteins were verified by colocalization evidence

**Figure 3:**
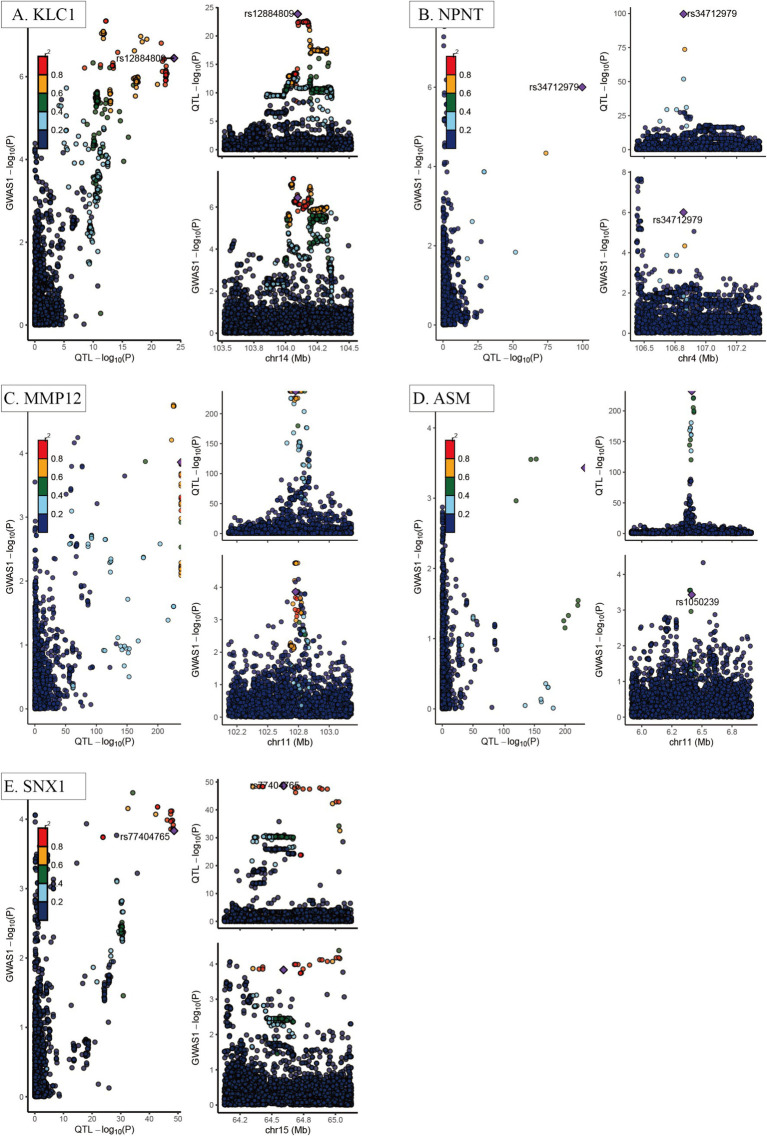
Co-localization analysis of key COPD-related proteins. **(A)** KLC1; **(B)** NPNT; **(C)** MMP12; **(D)** ASM; **(E)** SNX1. Each panel demonstrates the genomic regions where the causal variants for both the circulating proteins and COPD overlap, indicating a shared genetic basis, with strong colocalization evidence emphasized. The x-axis represents chromosome position (Mb), while the y-axis represents the −log10 (*p*-value) from GWAS or protein quantitative trait loci (pQTL) analysis results. Each dot represents an individual single nucleotide polymorphism (SNP), with color intensity indicating the strength of genetic association (greater than 0.75 indicates strong evidence of colocalization).

Of the eighteen proteins that were shown to be substantially related with COPD, five—KLC1, NPNT, MMP12, ASM, and SNX1—showed strong evidence of co-localization with genetic variations linked to COPD across distinct genomic windows (PP4 > 0.75) (Figure 3). This study used reverse MR analysis and negative control analysis to see if reverse causality or population structure affected the correlations between these proteins and COPD. There is extremely little chance of reverse causation or population structure bias, according to the data, which revealed no statistically significant relationships (*p* > 0.05).

### 3.3 Clinical plasma validation of COPD-related proteins

**Figure 4:**
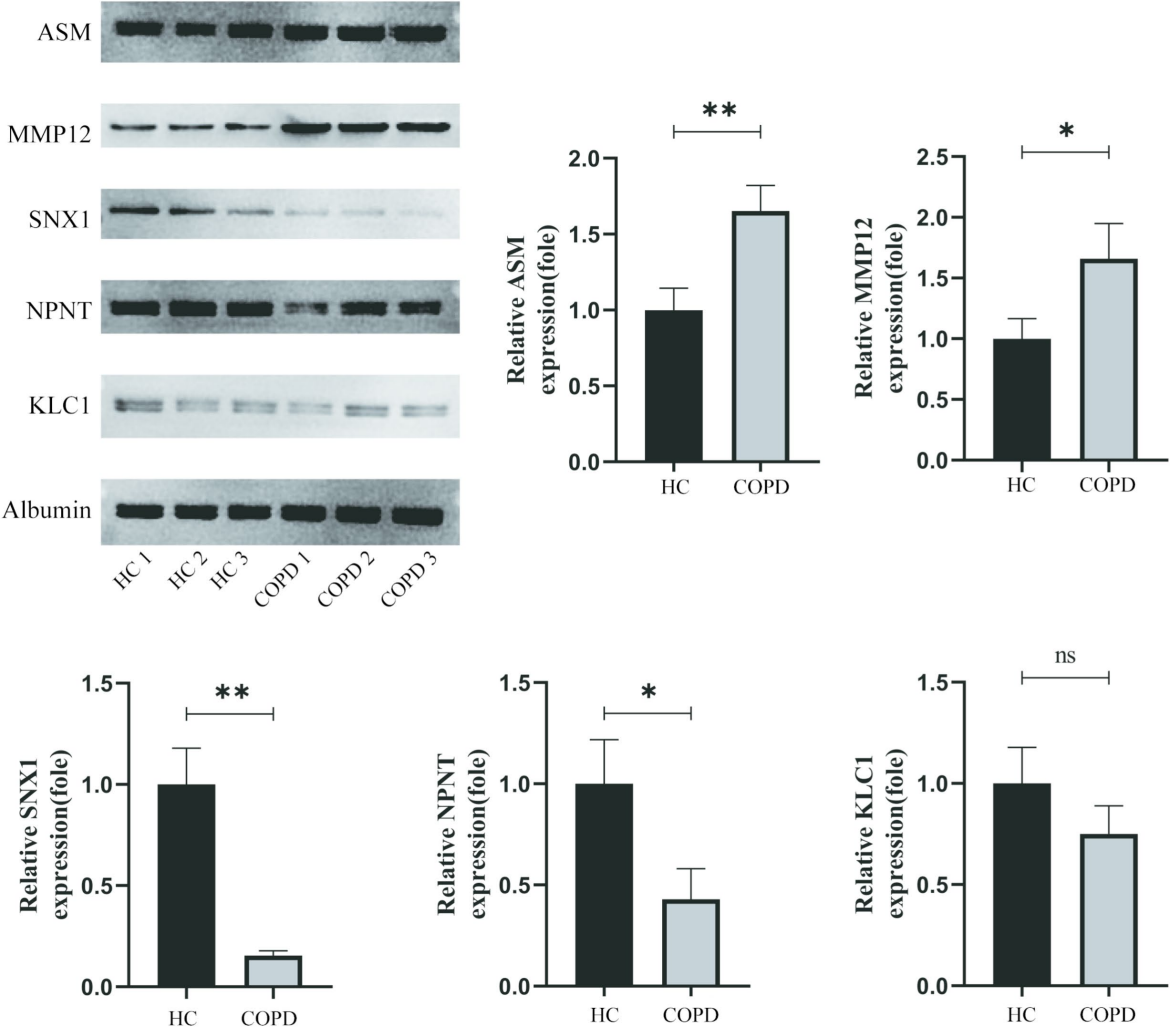
Western blot analysis of MMP12, ASM, KLC1, NPNT, and SNX1 expression levels in plasma samples from healthy controls (HC1–HC3) and COPD patients (COPD1–COPD3). Albumin was used as a loading control. Band intensities were quantified using ImageJ software. Statistical analysis was performed using Student’s t-test, with data presented as mean ± SEM (*p < 0.05).

Western blot analysis revealed significant differences in protein expression between the COPD and healthy control groups (Figure 4). Specifically, plasma levels of MMP12 and ASM were significantly elevated in the COPD group compared to controls (*p* < 0.05), while NPNT and SNX1 levels were significantly reduced (*p* < 0.05). No significant difference was observed in KLC1 expression between the two groups (*p* > 0.05). Albumin levels remained consistent across both groups, serving as a stable loading control.

### 3.4 Association of potential drug targets with current COPD medications

**Figure 5:**
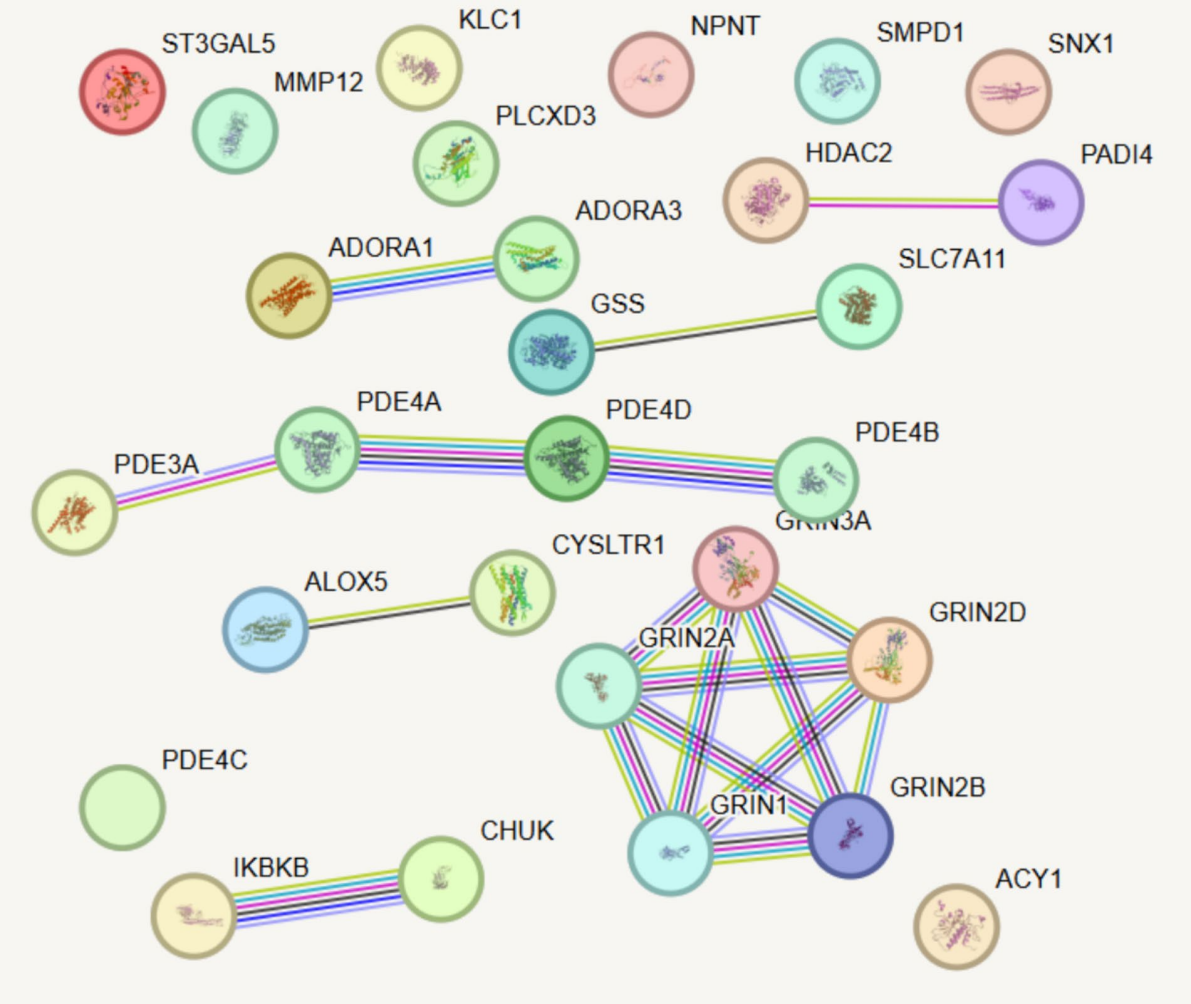
PPI network analysis integrating existing drug targets with newly identified candidate proteins associated with COPD. Line thickness indicates the predicted strength of protein–protein interactions, with thicker lines representing stronger interactions.

Six medications that are presently used to treat COPD were chosen in order to better investigate the possible impacts of current pharmacological therapy on the illness. These drugs are Roflumilast; Montelukast Sodium; Azithromycin; Aminophylline; Carbocisteine, and Acetylcysteine. They were selected due to their widely accepted clinical utility and proven ability to effectively treat COPD symptoms. Using a PPI network, this study further showed the targets of various medications and their possible interactions in this experiment by combining drug and protein target analysis. After examining how the six drug targets interacted with the five identified proteins, it was discovered that none of the drug targets significantly affected the outcomes pertaining to these proteins (Figure 5).

### 3.5 Healthy lifestyles prioritizing five proteins as interventional targets

**Figure 6:**
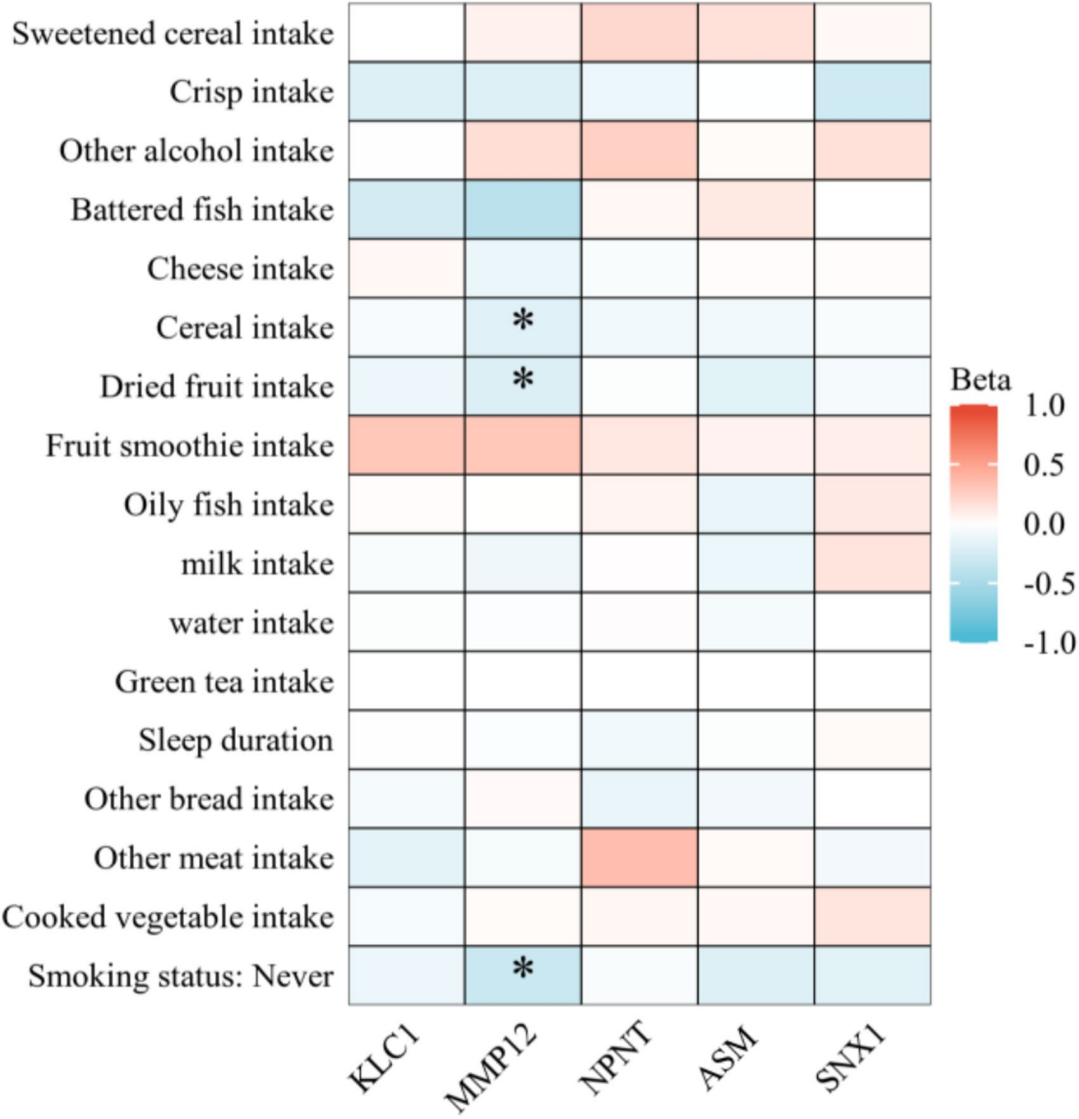
Heatmap illustrating the impact of various lifestyle factors on the expression of key COPD-related proteins. Heatmap colors represent the effect size (Beta value) of lifestyle factors on protein expression. Red indicates a positive effect (increased protein expression), while blue indicates a negative effect (decreased protein expression). Darker colors denote stronger statistical significance.

This examined the connections between 17 healthy lifestyle factors and five COPD-related proteins via a MR analysis. This study found many correlations between the proteins KLC1, MMP12, Nephronectin (NPNT), ASM, and SNX1 and other lifestyle factors (Figure 6). The expression of MMP12 was found to be significantly correlated with a number of lifestyle factors, including not smoking (*p* = 0.001878), nut consumption (*p* = 0.047915), and grain intake (*p* = 0.047915). It is possible that these dietary and lifestyle factors influence MMP12 expression, with smoking being associated with a notable increase in MMP12 expression. Higher *p*-values meant that KLC1, NPNT, and SNX1 did not exhibit significant relationships with the majority of lifestyle factors, although some linkages were still seen. For instance, SNX1 demonstrated mild correlations with smoking status and consumption of oily fish (both *p*-values greater than 0.2), whereas KLC1 revealed a substantial relationship in non-smokers (*p* = 0.580055). When it came to lifestyle factors, ASM showed lower connections than other proteins. Most *p*-values were more than 0.2, which means there were no significant differences.

## 4 Discussion

So far as we are aware, this is the first study to examine the causality between circulating proteins and COPD using both Bayesian colocalization analysis as well as two-sample MR. This study systematically investigated the causal connections between 4,907 circulating proteins and COPD, identifying 18 proteins connected to COPD susceptibility. This study prioritized five proteins—KLC1, NPNT, MMP12, ASM, and SNX1—that are linked to COPD via colocalization analysis. This study discovered during the druggability evaluation that several proteins have already been targeted for the treatment of COPD; Yet, no direct interactions were identified between the five selected COPD-related proteins and known drug targets. Furthermore, this study found that healthy lifestyle factors, especially MMP12, which demonstrated significant associations with smoking, grain intake, and nut consumption, can modulate the expression of these five proteins, suggesting new targets for the personalized treatment and early prevention of COPD.

First, our proteome-wide MR analysis of 4,907 circulating proteins revealed 18 proteins, including both protective and risk factors, that have a statistically significant causal association with COPD. Our findings align with prior studies suggesting that MMP12 contributes to tissue remodeling and inflammation in COPD, primarily through its role in elastin degradation (21). In contrast, ASM has been associated with oxidative stress and ceramide-induced apoptosis, supporting its involvement in chronic inflammation. Interestingly, NPNT, a protective protein, has been shown to play a role in lung development and epithelial cell integrity, which corroborates earlier findings. This study advances prior research by providing causal evidence through Bayesian colocalization, distinguishing it from correlative studies. NPNT, STAR5, α1-Antitrypsin, IL-17 RD, IL-1Ra, GOLM1, and SNX1 are among the proteins that have been found to lower the risk of COPD. Conversely, the following proteins have been linked to a higher risk of COPD: KLC1, Plasminogen, MSP, Angiostatin, sTie-1, PILRA isoform FDF03-deltaTM, PILRA isoform FDF03-M14, Cathepsin H, MMP12, ASM, and COAA1. To minimize bias from horizontal pleiotropy, this study exclusively used cis-pQTLs as instruments, as they directly influence the transcription and/or translation of related genes (22). Additionally, bias caused by LD was eliminated using Bayesian colocalization analysis. KLC1, NPNT, MMP12, ASM, and SNX1 are five COPD-related proteins that were highly confidently identified with a posterior probability threshold of 0.75. This suggests that these proteins could be important for the development and progression of COPD. These results reveal that early blood testing might be used in clinical settings to predict the progression of illness. These proteins also show promise as potential targets for the creation of novel COPD therapies, which may enhance the disease outcomes as well as therapeutic efficacy.

To further strengthen the causal inference derived from Mendelian randomization (MR) analyses, we performed Western blot validation of key proteins in an independent clinical cohort. The results revealed significantly elevated plasma levels of MMP12 and ASM, and significantly reduced levels of NPNT and SNX1 in COPD patients compared to healthy controls (Figure 4), consistent with directions predicted by genetic analyses. These experimental findings provide further support for the pathological roles of MMP12 and ASM as risk factors, and NPNT and SNX1 as protective factors in COPD. Notably, no significant difference in KLC1 expression was observed between groups (*p* > 0.05). This discrepancy could be attributed to ethnic differences between populations (the MR analysis was based on European ancestry data, whereas the clinical cohort was Asian), or due to mechanisms such as tissue-specific regulation or post-translational modifications not captured by plasma proteomics. These factors warrant further investigation. Overall, the consistency between genetic predictions and clinical protein measurements underscores the translational value of this study in bridging population genetic evidence and molecular phenotypic associations.

The metalloproteinase MMP12, which is released by macrophages, contributes to tissue remodeling and airway inflammation in people with COPD. Oxidative stress induced by smoking may exacerbate lung tissue damage and inflammation by activating MMP12 and ASM through pro-inflammatory signaling cascades. Additionally, dietary intake of foods such as nuts and grains may regulate the expression of MMP12 by modulating macrophage activity and cytokine production (23, 24). Its overexpression causes the breakdown of lung tissue structure, especially elastin degradation, which is directly related to the development of emphysema (25, 26). It also breaks down elastin in the extracellular matrix. This is consistent with our finding that MMP12 poses a substantial risk for COPD. Additionally, MMP12 may be a potential therapeutic target for COPD since animal studies have demonstrated that blocking MMP12 can significantly ameliorate the morphological alterations and functional deficits linked to emphysema and small airway remodeling (27, 28). Furthermore, MMP12 is intimately linked to both chronic inflammation and fibrosis in addition to its role in the degradation of elastin, which adds to the reduction in lung function. Research has indicated that inhibiting MMP12 activity holds potential for slowing COPD progression, particularly in reducing emphysema and airway remodeling. As a result, medicines that target MMP12 inhibition are presently being investigated in relevant clinical studies (26). MMP12 levels are markedly raised in COPD patients due to smoking, according to research conducted recently. It has been discovered that smoking increases the risk of COPD development by 4.5–6 times in those with the MMP12 T/T genotype (29). This agrees with what we found. Clinically speaking, it’s critical to inform patients with noticeably raised MMP12 levels that smoking may hasten the progression of COPD and that early intervention can help to delay the disease’s progression.

The hydrolysis of sphingomyelin into ceramide (Cer) is catalyzed by the sphingolipid metabolism enzyme ASM (acid sphingomyelinase). Ceramide acts as a lipid second messenger, regulating cell growth and apoptosis. Hence, ASM plays a crucial role in processes like apoptosis, immune function, as well as inflammation (30). Lea et al. (31) discovered that COPD patients’ alveolar macrophages have noticeably higher expression levels of the NSM-2 and ASM proteins. Elevated Cer levels can be efficiently decreased by inhibiting ASM and/or NSM-2. Thus, modulating and restoring the normal function of macrophages through the inhibition of ASM and/or NSM-2 could be beneficial in the treatment of COPD. This study also found a significant positive correlation between ASM protein levels and COPD. Recent research shows that smoking-induced oxidative stress activates ASM, triggering inflammatory cell recruitment and cytokine release, worsening chronic lung inflammation (32). The production of circulating endothelial microparticles containing different microRNAs by ASM can be triggered by cigarette smoke. This can then impact the capacity of specialized macrophages to eliminate apoptotic cells. These targeted effects may be critical in the pathophysiology of smoking-related illnesses including endothelial damage as well as inflammation (33). Because of this, ASM is thought to be a viable therapeutic target for COPD, and preventing its action might aid in lowering inflammation and delaying the progression of the illness.

The primary function of KLC1, a crucial member of the kinesin light chain family, is intracellular trafficking. Its light chain is essential to these activities because it binds to several proteins to function as an adapter molecule. Through its interactions with other proteins, KLC1 also plays a role in the development and progression of a number of cancers as well as neurodegenerative illnesses (34–36). For instance, it has been demonstrated that drug resistance and lung cancer metastasis are significantly influenced by the interaction between KLC1 and ALK (37, 38). Nevertheless, not much research has been done on the direct connection between KLC1 and COPD. Apoptosis, oxidative stress, as well as chronic inflammation are all involved in the pathophysiology of COPD; these processes may be directly related to anomalies in intracellular transport. This presents a possible direction for further in-depth investigation.

NPNT and SNX1 were identified in this study as protective proteins against COPD. NPNT is a novel extracellular matrix protein involved in various biological functions such as cell proliferation, adhesion, and differentiation. According to earlier research, alveolar epithelial cells and fetal and adult lung tissues both exhibit significant levels of NPNT expression, which may have an impact on lung development and function (39, 40). Consistent with our findings, Qian et al. (41) also found that NPNT expression is considerably downregulated in COPD patients’ lung tissues. Consequently, this study hypothesize that NPNT activation might aid in delaying the progression of COPD. The main roles of the protein SNX1, which is found in the Golgi apparatus and endoplasmic reticulum, are in vesicle trafficking and cellular secretion. As a potential tumor suppressor, SNX1 is crucial in the development and progression of cancers. For instance, studies has demonstrated that SNX1 expression is dramatically decreased in colorectal as well as non-small cell lung cancer cells (42, 43), and it is crucial to the pathogenesis of these tumors. Research has demonstrated that overexpression of SNX1 can decrease the amount of epidermal growth factor receptors (EGFR) on the cell surface (44), thereby inhibiting EGFR activation and supporting the integrity of the airway epithelial barrier (45), even though no *in vivo* or *in vitro* experiments have yet established a direct link between SNX1 and COPD. Our results lead us to hypothesize that SNX1 may represent yet another crucial COPD treatment target.

Furthermore, this study examined the targets of different COPD drugs and their possible interrelationship by integrating the PPI network in the evaluation of drug targets. For instance, Roflumilast primarily targets the PDE4 protein family, which is consistent with previous research findings (46–48). Moreover, the PPI network revealed complex interactions between Roflumilast and the targets of other COPD-related drugs, providing valuable insights for new drug development and combination therapy strategies. Montelukast Sodium and Azithromycin also demonstrated unique target mechanisms, further elucidating the interaction network of existing drugs. Notably, none of the identified proteins overlap with current COPD drug targets, underscoring the novelty of these findings. For instance, targeting MMP12, which plays a critical role in tissue remodeling, could complement existing therapies aimed at alleviating symptoms rather than addressing underlying molecular mechanisms. This suggests a promising avenue for developing therapies that specifically inhibit MMP12 to slow disease progression.

Eventually, this study also explored the influence of lifestyle factors on the regulation of these proteins, finding that a healthy lifestyle has a significant impact on the expression of COPD-related proteins. For instance, MMP12 expression was highly correlated with the consumption of grains, nuts, and the status of not smoking, indicating that dietary habits and quitting smoking may affect the progression of COPD by modifying MMP12 expression (29). Despite the lack of substantial relationships between lifestyle factors and KLC1, Nephronectin, and SNX1, several correlations were nevertheless identified, suggesting that lifestyle decisions may have some impact on their expression. MMP12, for example, was significantly influenced by smoking status and dietary habits. Lifestyle modifications, such as smoking cessation and dietary changes, may influence COPD-related protein expression. Higher intake of grains and nuts was linked to reduced MMP12 levels, potentially mitigating inflammation and disease progression. Combined with pharmacological treatments, these interventions could enhance COPD prevention and management. Interestingly, KLC1 exhibited a moderate association in non-smokers, implying that this protein may have some regulatory potential in response to environmental and behavioral interventions.

There are several limitations on this study. First, the findings might not apply to other groups since the sample population was limited to those of European ancestry. Second, our analysis of six established COPD treatments revealed no interactions with the five COPD-related proteins identified through colocalization, suggesting that future studies should expand the range of drug target testing. Third, this study lacked direct experimental validation of the identified protein targets. Future research should include *in vitro* and *in vivo* studies on proteins such as MMP12 and ASM to elucidate their precise molecular roles in COPD pathogenesis and to evaluate their potential as therapeutic targets. Fourth, despite the drawbacks of MR, such as the possibility of bias from weak instruments as well as horizontal pleiotropy, the instruments employed in this investigation had high F-statistics and no discernible pleiotropy, thereby reducing these biases to the greatest extent. Furthermore, it is still difficult to completely eliminate confounders even if the MR design aims to minimize confounding as well as reverse causation, and reverse MR analysis revealed no evidence of reverse causality. In order to confirm these results, larger-scale epidemiological studies and experimental investigations should be performed in the future to better assess the association between plasma protein concentrations and COPD.

## 5 Conclusion

In conclusion, this study identifies 18 circulating proteins associated with COPD through proteome-wide Mendelian randomization. Among these, five key proteins—KLC1, NPNT, MMP12, ASM, and SNX1—were prioritized based on colocalization analysis (PP4 > 0.75), The identification of MMP12, ASM, and other key proteins offers insights for COPD management. These proteins are potential biomarkers for early diagnosis and targets for therapeutic intervention. Integrating lifestyle modifications, such as dietary changes and smoking cessation, could enhance prevention strategies. Offering novel proof of possible drug targets and lifestyle regulating factors. In order to provide fresh perspectives and approaches for the early detection, therapeutic interventions, and prevention of COPD, future studies should delve further into the specific mechanisms of these proteins, particularly those pertaining to high-risk factors like MMP12, KLC1, and ASM.

## Data Availability

The original contributions presented in the study are included in the article/Supplementary material, further inquiries can be directed to the corresponding authors.

## Footnotes

^1^ https://string-db.org

^2^ https://go.drugbank.com
