# Supplementary material for: Proteomic insights into COPD pathogenesis and therapeutic targets: a causal analysis of circulating proteins
Source: Front Med (Lausanne). 2025 May 12;12:1529495. doi: 10.3389/fmed.2025.1529495 (PMC12104083; doi:10.3389/fmed.2025.1529495)
Supplement: Supplementary file 1 [file Table_1.DOCX]

**Supplement Table 1 MR results for plasma proteins signiffcantly associated with COPD after Bonferroni correction**

| Protein | SNP | Effect allele | OR (95% CI) b | P value | FDR |
| --- | --- | --- | --- | --- | --- |
| KLC1 | rs12884809 | A | 1.81（1.44-2.27） | 3.57*10^-7^ | <0.05 |
| Nephronectin | rs34712979 | A | 0.75（0.67-0.84） | 9.97*10^-7^ | <0.05 |
| Plasminogen | rs11751347  rs4252159  rs4252185  rs6938647 | T  A  C  A | 1.23(1.13-1.34) | 2.19*10^-6^ | <0.05 |
| MSP | rs2230169  rs1131095 | G  C | 1.04(1.02-1.06) | 1.48*10^-5^ | <0.05 |
| Angiostatin | rs11751347  rs1652492  rs4252185 | T  A  C | 1.20(1.11-1.31) | 1.55*10^-5^ | <0.05 |
| sTie-1 | rs7549876 | T | 1.29(1.14-1.45) | 4.06*10^-5^ | <0.05 |
| STAR5 | rs11634869  rs28524742  rs4392019 | T  G  T | 0.85(0.78-0.92) | 4.58*10^-5^ | <0.05 |
| a1-Antitrypsin | rs1243167  rs17580  rs28929474  rs709932 | A  A  T  T | 0.87(0.81-0.93) | 4.43*10^-5^ | <0.05 |
| IL-17 RD | rs6809523 | G | 0.82(0.75-0.91) | 8.09*10^-5^ | <0.05 |
| PILRA isoform FDF03-deltaTM | rs1859788 | A | 1.03(1.02-1.05) | 1.88*10^-4^ | <0.05 |
| PILRA isoform FDF03-M14 | rs1859788 | A | 1.03(1.02-1.05) | 1.88*10^-4^ | <0.05 |
| Cathepsin H | rs62013199 | G | 1.13(1.06-1.20) | 1.54*10^-4^ | <0.05 |
| IL-1Ra | rs55709272  rs6743376 | C  C | 0.83(0.75-0.91) | 1.73*10^-4^ | <0.05 |
| GOLM1 | rs117508530  rs4333693 | T  A | 0.85(0.79-0.93) | 1.67*10^-4^ | <0.05 |
| MMP-12 | rs117698348  rs28381684  rs4085170 | G  T  G | 1.07(1.03-1.11) | 2.23*10^-4^ | <0.05 |
| SNX1 | rs2166735  rs77404765 | T  C | 0.77(0.67-0.88) | 2.12*10^-4^ | <0.05 |
| ASM | rs1050239 | A | 1.23(1.10-1.38) | 3.70*10^-4^ | <0.05 |
| COAA1 | rs9488842 | C | 1.19(1.08-1.32) | 5.55*10^-4^ | <0.05 |
